# Supplementary material for: Experimental Conditions That Influence the Utility of 2′7′-Dichlorodihydrofluorescein Diacetate (DCFH2-DA) as a Fluorogenic Biosensor for Mitochondrial Redox Status
Source: Antioxidants (Basel). 2022 Jul 22;11(8):1424. doi: 10.3390/antiox11081424 (PMC9329753; doi:10.3390/antiox11081424)
Supplement: Supplementary file 1 [file antioxidants-11-01424-s001.zip › antioxidants-1779860-supplementary.pdf]

antioxidants

MDPI

Article

Experimental Conditions That Influence the Utility of 2′7′-Dichlorodihydrofluorescein Diacetate ($DCFH_2$-DA) as a Fluorogenic Biosensor for Mitochondrial Redox Status

Lianne R. de Haan [1,2,†], Megan J. Reiniers [1,3,4,†], Laurens F. Reeskamp [5], Ali Belkouz [6], Lei Ao [1], Shuqun Cheng [7], Baoyue Ding [1,‡], Rowan F. van Golen [8,‡] and Michal Heger [1,2,4,9,‡,*]

1 Jiaxing Key Laboratory for Photonanomedicine and Experimental Therapeutics, Department of Pharmaceutics, College of Medicine, Jiaxing University, Jiaxing 314001, China; l.dehaan@erasmusmc.nl (L.R.d.H.); m.reiniers@haaglandenmc.nl (M.J.R.); aolei@zjxu.edu.cn (L.A.); lena_310@zjxu.edu.cn (B.D.)
2 Laboratory for Experimental Oncology, Department of Pathology, Erasmus MC, 3015 GD Rotterdam, The Netherlands
3 Department of Surgery, Haaglanden Medisch Centrum, 2262 BA The Hague, The Netherlands
4 Membrane Biochemistry and Biophysics, Department of Chemistry, Faculty of Science, Utrecht University, 3584 CH Utrecht, The Netherlands
5 Department of Vascular Medicine, Amsterdam University Medical Centers, Location AMC, 1105 AZ Amsterdam, The Netherlands; l.f.reeskamp@amsterdamumc.nl
6 Department of Medical Oncology, Amsterdam University Medical Centers, Location AMC, University of Amsterdam, Cancer Center Amsterdam, 1105 AZ Amsterdam, The Netherlands; a.belkouz@amsterdamumc.nl
7 Department of Hepatic Surgery VI, The Eastern Hepatobiliary Surgery Hospital, The Second Military Medical University, Shanghai 200438, China; chengshuqun@smmu.edu.cn
8 Department of Gastroenterology and Hepatology, Leiden University Medical Center, 2333 ZA Leiden, The Netherlands; r.f.van_golen@lumc.nl (R.F.v.G.)
9 Department of Pharmaceutics, Utrecht Institute for Pharmaceutical Sciences, Utrecht University, 3584 CG Utrecht, The Netherlands
* Correspondence: m.heger@jctres.com or m.heger@uu.nl (M.H.); Tel.: +31-6-24483083 (M.H.) or +31-30-2533966 (M.H.)
† These authors contributed equally to the work.
‡ Shared senior authorship.

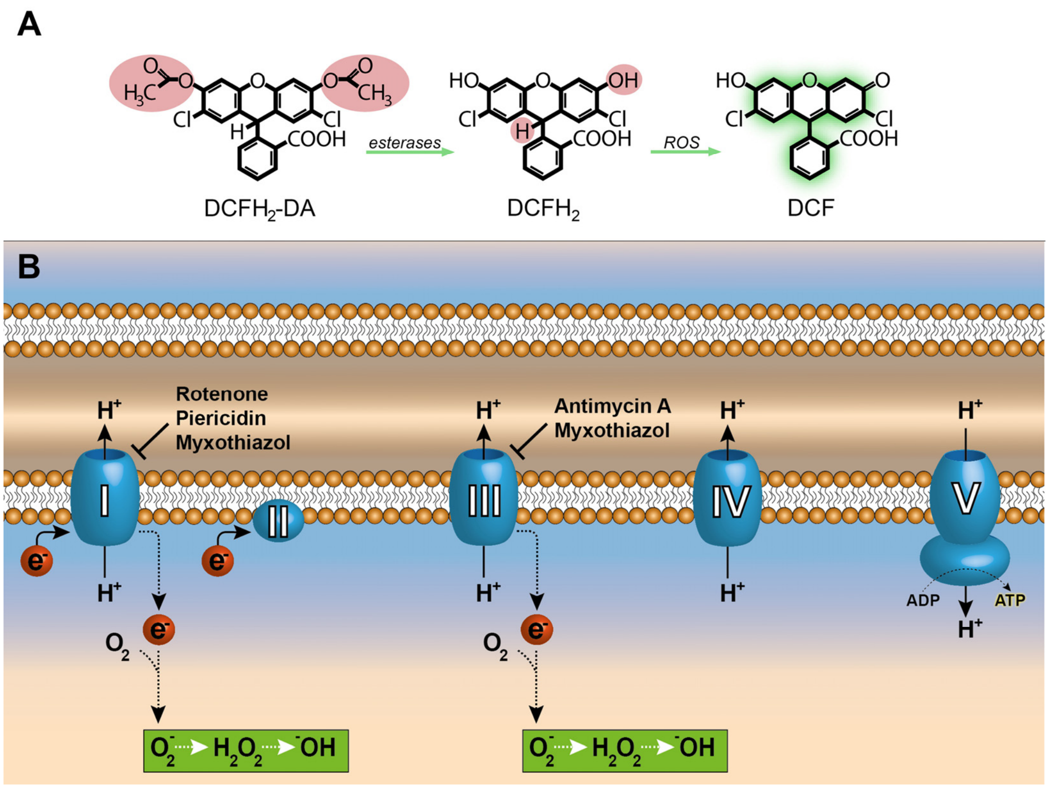

Figure S1. Reaction scheme of DCFH2-DA to DCF (A) and a simplified overview of the electron transport chain and used inhibitors (B).

Table S1. List of chemicals and reagents.

Compound	Purity	Supplier	Additional information
Accutase	n/a	Innovative Cell Technologie[€]	
Accumax	n/a	Innovative Cell Technologies[€]	
Antimycin A	n/a	Sigma-Aldrich[#]	
bovine serum albumin (BSA),	≥ 96%	Sigma-Aldrich[#]	
2'7'-dichlorodihydrofluorescein diacetate ($DCFH_2$-DA)	n/a	Life Technologies[)]	
Dihydrorhodamine 123	n/a	Life Technologies[)]	
DMSO	≥ 99.5%	Merck Millipore[*]	
DNA stain Hoechst 33342	n/a	ImmunoChemistry Technologies[(]	
Dulbecco's modified Eagle medium	n/a	Lonza[†]	Phenol red-free
Dulbecco's modified Eagle medium/Ham's F12	n/a	Hyclone[!]	1:1 volume ratio
Ethanol absolute (CH_3CH_2OH)	≥ 99.5%	J.T. Baker[&]	

Fetal calf serum (FCS)	n/a	Bodinco?	
4-(2-hydroxyethyl)-1-piperazineethanesulfonic acid (HEPES)	≥ 99%	Merck Millipore*	
Human recombinant insulin solution	≥ 27 U/mg	Sigma-Aldrich#	
Hydrocortisone 21-hemisuccinate	n/a	Sigma-Aldrich#	
Myxothiazol	≥ 98%	Sigma-Aldrich#	
L-glutamine	n/a	Lonza†	
Methanol (MeOH)	≥ 99%	Alfa Aesar$	
MitoSOX	n/a	Life Technologies)	
Rotenone	≥ 95%	Sigma-Aldrich#	
tris(hydroxymethyl)aminomethane (TRIS),	≥ 99.8%	Sigma-Aldrich#	
Penicillin/streptomycin	n/a	Lonza†	
Sterile phosphate-buffered saline	n/a	Fresenius Kabi§	
Piericidin A	≥ 95%	Santa Cruz Biotechnology+	
Roswell Park Memorial Institute medium 1640 (RPMI-1640)	n/a	Lonza†	Phenol red-free
Water	Milli-Q	Merck Millipore&	18.2 MΩ · cm at 25 °C
William's E medium	n/a	Lonza†	Phenol red-free
William's E medium	n/a	Lonza†	With phenol red
William's E medium	n/a	MT-diagnostics^	Without pyruvate and glucose

€ San Diego, CA

St. Louis, MO

) Carlsbad, CA

* Darmstadt, Germany

(Alkmaar, the Netherlands

† Basel, Switzerland

! South Logan, UT

& Center Valley, PA

? Bloomington, MN

$ Karlsruhe, Germany

§ Graz, Austria

+ Santa Cruz, CA

^ Etten-Leur, the Netherlands

Table S2. Mitochondrial inhibitor concentrations and incubation times used in published protocols.

Rotenone

Ref	Concentration(s) used [µM]	Incubation time [h]	Cells | cell lines
[1]	2	24	Human hepatocytes (obtained during liver transplantation)

[2]	6.3 ;25	24 ;48; 72	HepG2
[3]	0.1; 0.5; 1.0; 2.0; 5.0	24 ;48; 72	HepG2
[4]	0.001; 0.01; 0.1; 1; 10; 100	24	HepG2
[5]	0.5	24	HepG2
[6]	12.5; 25; 50; 100; 250	24	HepG2
[7]	0.1	18	Sk-Hep-1
[8]	0.5; 1; 1.5; 2.0; 2.5; 5; 7.5; 10	4; 6; 12; 24	WB-F344

Antimycin A

Ref	Concentration(s) used [µM]	Incubation time [h]	Cells | organelles
[9]	15	Not listed	Isolated rat liver mitochondria
[10]	15	0; 6; 12; 24	Rat hepatocytes
[11]	20	1	Eel hepatocytes
[12]	200	2	Rat hepatocytes
[13]	5	0-4	Rat hepatocytes
[14]	10	Continuous	Rat subsarcolemmal mitochondria
[15]	10	Continuous	Heart mitochondria of pigeons and rats
[16]	1	Continuous	Isolated rat brain mitochondria

Myxothiazol

Ref	Concentration(s) used [µM]	Incubation time [h or min]	Cells | organelles
[17]	3.0; 1.5	"Several min"	Beef and sheep heart submitochondrial particles
[18]	4	8 h	Mouse muscle cells
[11]	5	5 h	Eel hepatocytes
[19]	0.6; 1.0; 5.0; 10.0	5; 15; 30; 60 min	Rat hepatocytes
[20]	10	3 min	Submitochondrial particles of rat hepatocytes
[21]	0-3.0	20 min	Rat hepatocytes

Piericidin A

Ref	Concentration(s) used [µM]	Incubation time [h]	Cells | organelles
[22]	0.2	Continuous	Isolated mitochondria bovine heart
[23]	2	Continuous	Rat skeletal muscle mitochondria
[24]	0-0.050	9	Mouse neurons
[25]	1	Continuous	Bovine heart mitochondria

[26]	1	Continuous	Bovine heart mitochondria

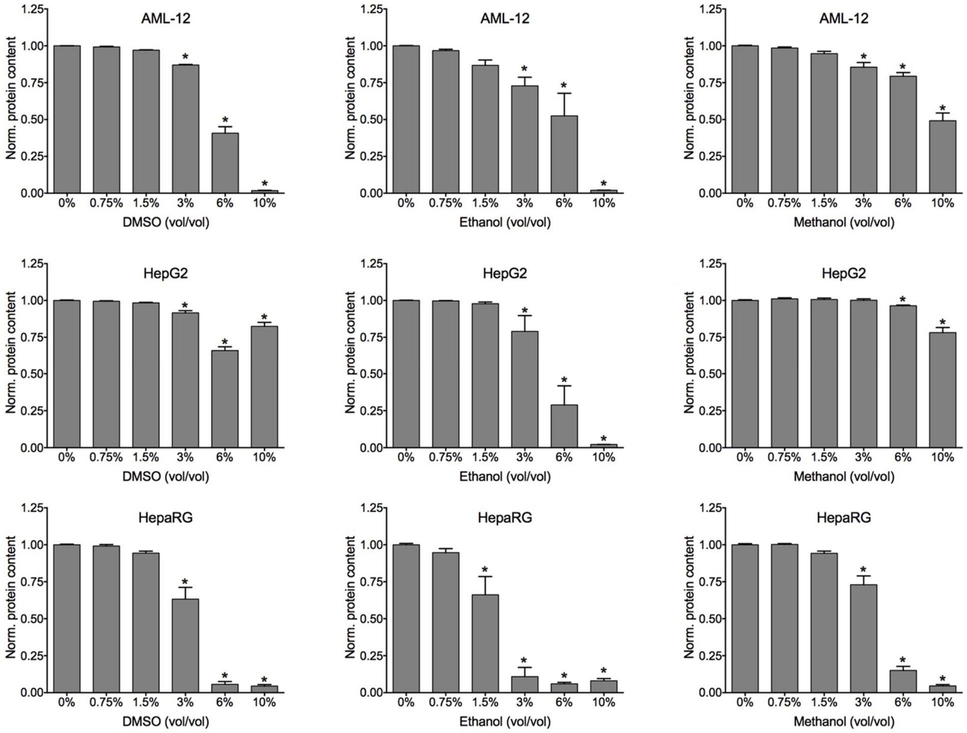

Figure S2. Solvent toxicity in hepatocyte cell lines. AML-12, HepG2, and HepaRG cells were cultured as described in the main manuscript. Directly (AML-12, HepG2) or 4 weeks (HepaRG) after reaching confluence, the monolayer was washed with 1 mL of PBS, after which the cells received fresh culture medium containing 0 – 10% (vol/vol) of ethanol, DMSO, or methanol. Following 72 h of incubation, the protein content per well was determined as a measure for solvent toxicity using the sulforhodamine B assay according to [27]. The results were normalized to the protein content of the control wells (i.e., 0% solvent) and are presented as mean ± SD (n=4 per concentration). Intragroup differences were tested using a one-way ANOVA with Dunnett's multiple comparison test, where * indicates $p < 0.05$ versus the 0% solvent group.

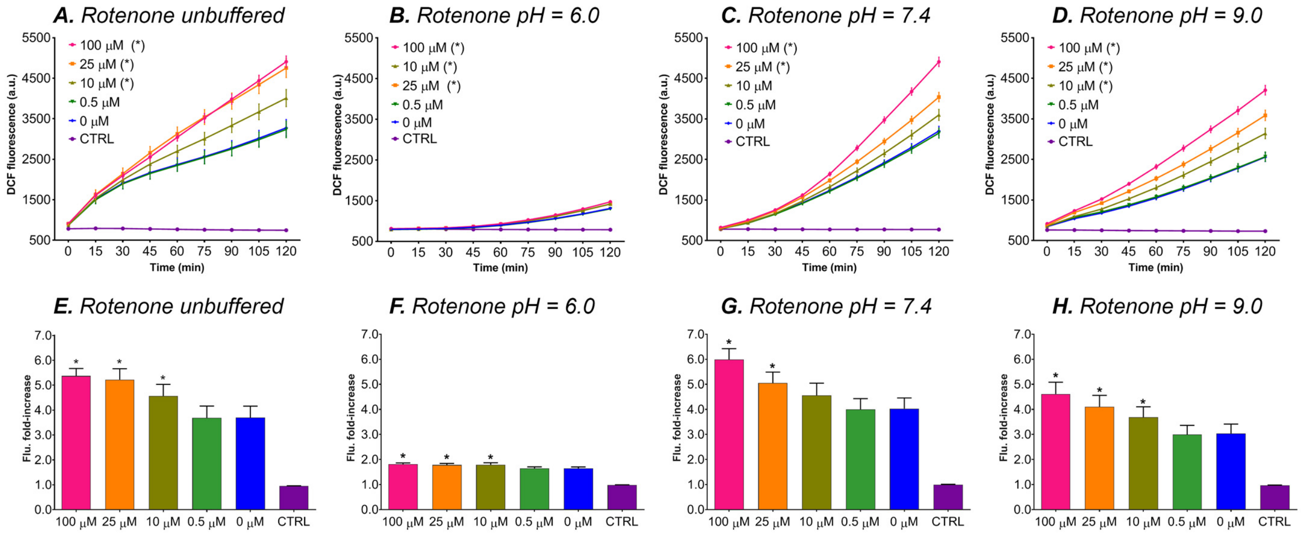

Figure S3. Influence of rotenone on DCF formation under cell-free conditions in unbuffered assay medium (**A** and **E**) or in assay medium buffered with 25 mM HEPES set to pH = 6.0 (**B** and **F**), pH = 7.4 (**C** and **G**), or pH = 9.0 (**D** and **H**) (i.e., the data summarized in Table 1 of the main manuscript). DCF fluorescence was recorded for 2 h using a microplate reader and is presented as mean ± SEM for 8 values per condition. Additional experimental details are provided in the main manuscript (see *Section 3.1* and the Table 1 legend). The mean (± SD) fold-increase in DCF fluorescence over 2 h is presented in panels (**E-H**) for clarity. For all shown experiments, * indicates $p < 0.05$ versus the 0 μM (i.e., vehicle control) group. Statistical analysis was performed as described in the main manuscript (see *Section 2.6* and Table 1 legend).

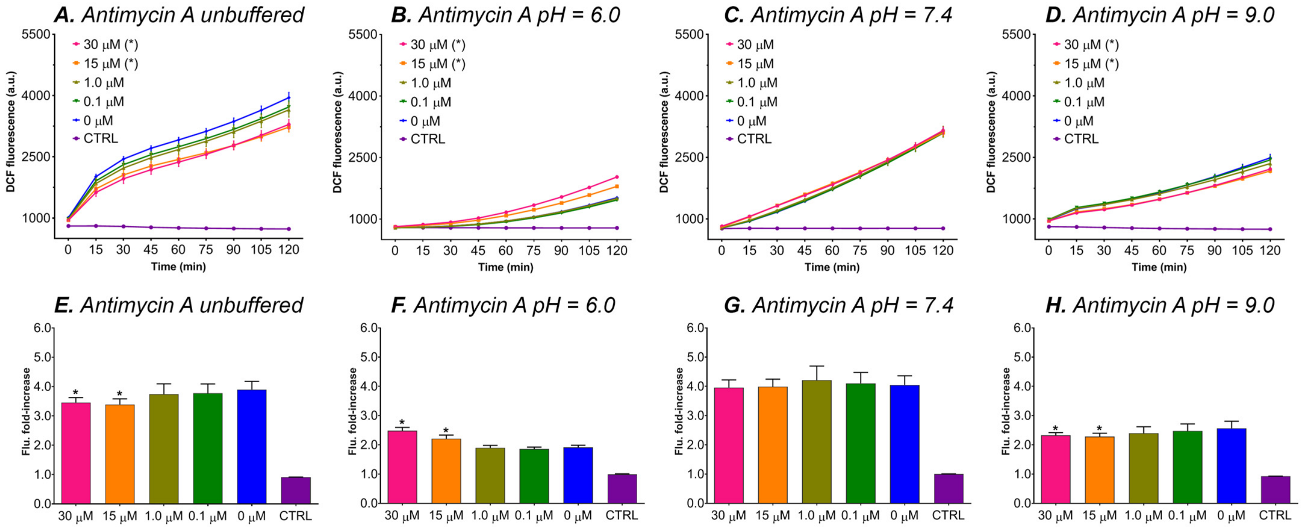

Figure S4. Influence of antimycin A on DCF formation under cell-free conditions in unbuffered assay medium (**A** and **E**) or in assay medium buffered with 25 mM HEPES set to pH = 6.0 (**B** and **F**), pH = 7.4 (**C** and **G**), or pH = 9.0 (**D** and **H**) (i.e., the data summarized in Table 1 of the main manuscript). DCF fluorescence was recorded for 2 h using a microplate reader and is presented as mean ± SEM for 8 values per condition. Additional experimental details are provided in the main manuscript (see *Section 3.1* and the Table 1 legend). The mean (± SD) fold-increase in DCF fluorescence over 2 h is presented in panels (**E-H**) for clarity. For all shown

experiments, * indicates p < 0.05 versus the 0 μM (i.e., vehicle control) group. Statistical analysis was performed as described in the main manuscript (see *Section 2.6* and Table 1 legend).

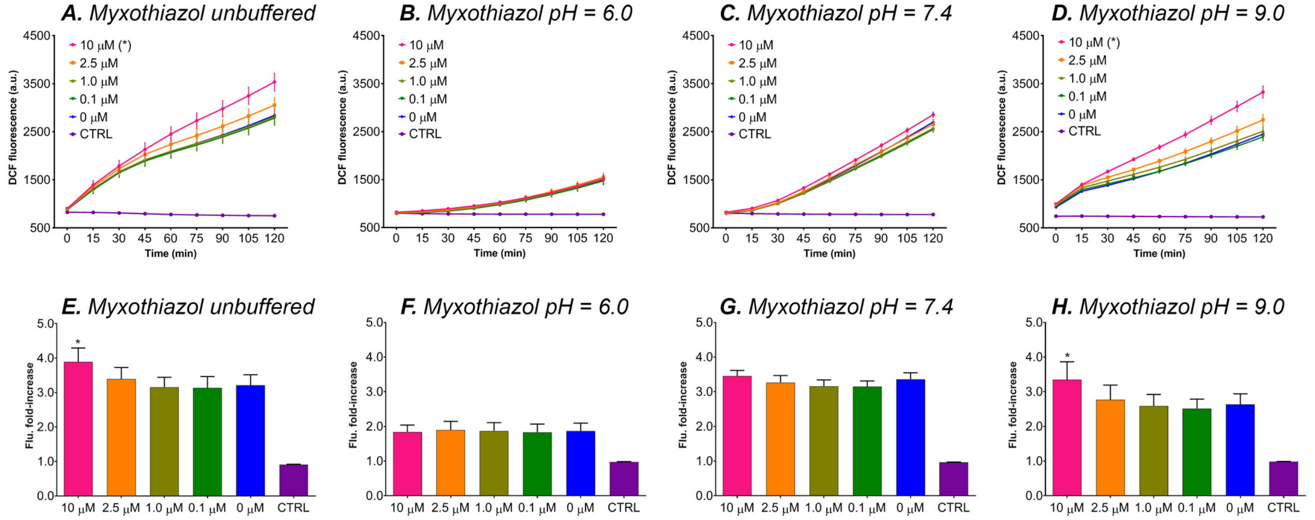

Figure S5. Influence of myxothiazol on DCF formation under cell-free conditions in unbuffered assay medium (**A** and **E**) or in assay medium buffered with 25 mM HEPES set to pH = 6.0 (**B** and **F**), pH = 7.4 (**C** and **G**), or pH = 9.0 (**D** and **H**) (i.e., the data summarized in Table 1 of the main manuscript). DCF fluorescence was recorded for 2 h using a microplate reader and is presented as mean ± SEM for 8 values per condition. Additional experimental details are provided in the main manuscript (see *Section 3.1* and the Table 1 legend). The mean (± SD) fold-increase in DCF fluorescence over 2 h is presented in panels (**E-H**) for clarity. For all shown experiments, * indicates p < 0.05 versus the 0 μM (i.e., vehicle control) group. Statistical analysis was performed as described in the main manuscript (see *Section 2.6* and Table 1 legend).

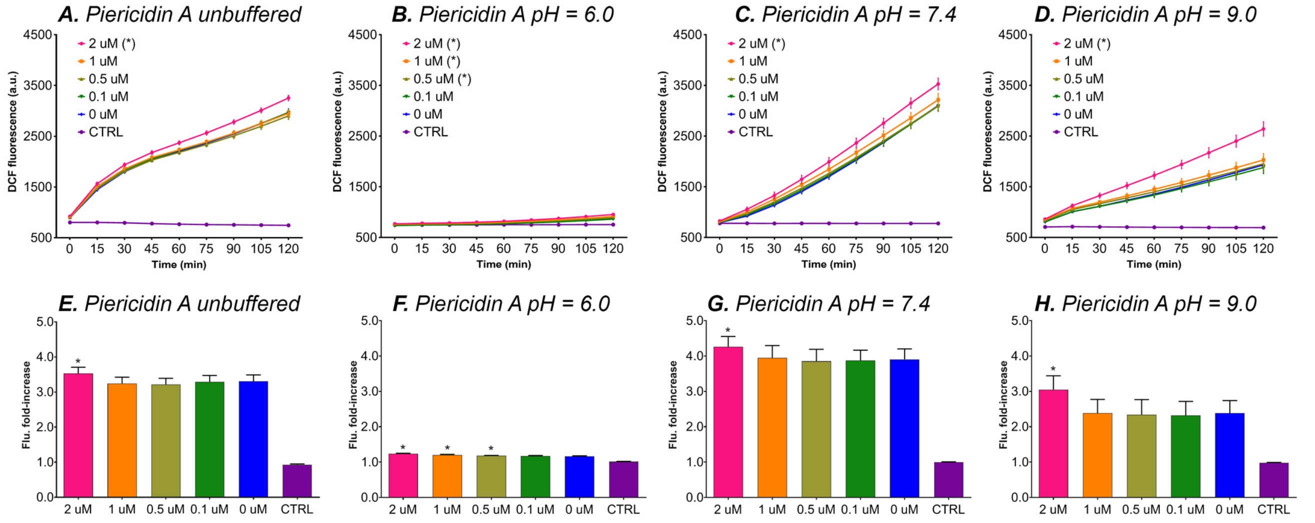

Figure S6. Influence of antimycin A on DCF formation under cell-free conditions in unbuffered assay medium (**A** and **E**) or in assay medium buffered with 25 mM HEPES set to pH = 6.0 (**B** and **F**), pH = 7.4 (**C** and **G**), or pH = 9.0 (**D** and **H**) (i.e., the data summarized in Table 1 of the main manuscript). DCF fluorescence was recorded for 2 h using a microplate reader and is presented as mean ± SEM for 8 values per condition. Additional experimental details are provided in the main manuscript (see *Section 3.1* and the Table 1 legend). The mean (± SD) fold-increase in DCF fluorescence over 2 h is presented in panels (**E-H**) for clarity. For all shown experiments, * indicates p < 0.05 versus the 0 μM (i.e., vehicle control) group. Statistical analysis was performed as described in the main manuscript (see *Section 2.6* and Table 1 legend).

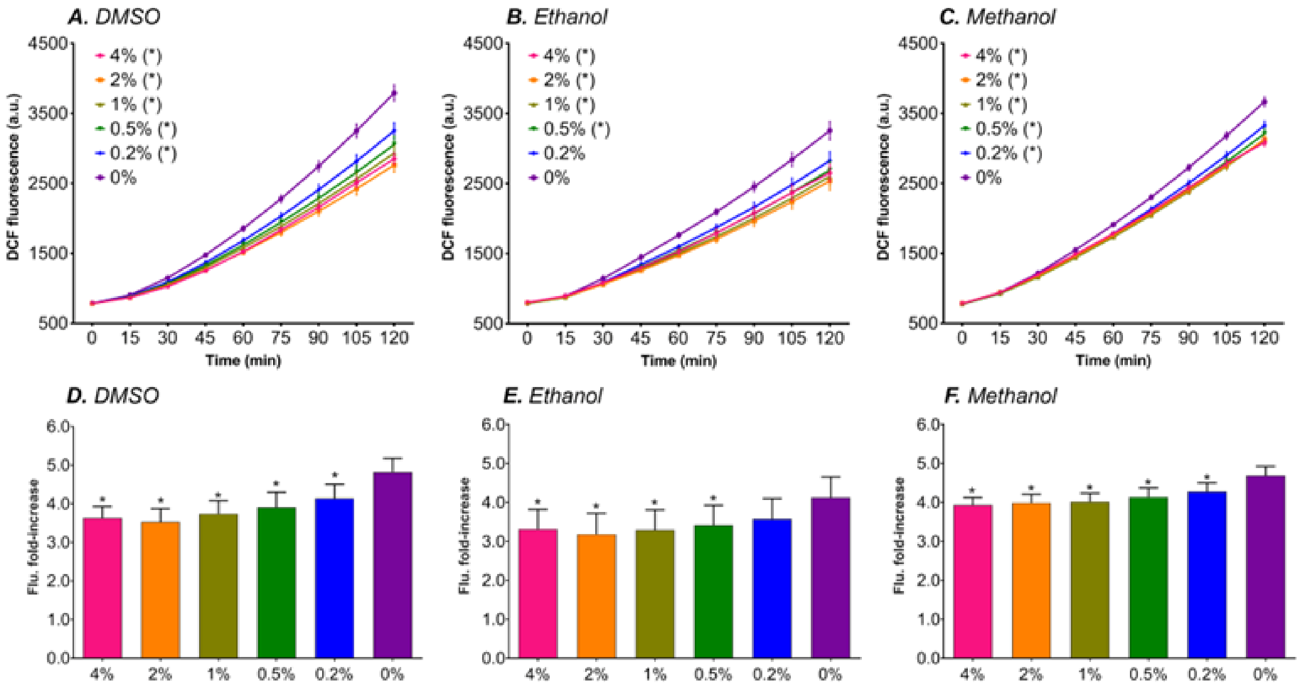

Figure S7. Influence of solvents on DCF formation under cell-free conditions in DMSO (**A** and **D**), ethanol (**B** and **E**), and methanol (**C** and **F**) in milliQ buffered with 25 mM HEPES set to pH = 7.4 (i.e., the data summarized in Table 2 of the main manuscript). DCF fluorescence was recorded for 2 h using a microplate reader and is presented as mean ± SEM for 8 values per condition. Additional experimental details are provided in the main manuscript (see *Section 3.1* and the Table 1 legend). The mean (± SD) fold-increase in DCF fluorescence over 2 h is presented in panels (**D-F**) for clarity. For all shown experiments, * indicates $p < 0.05$ versus the 0 μM (i.e., vehicle control) group. Statistical analysis was performed as described in the main manuscript (see *Section 2.6* and Table 2 legend).

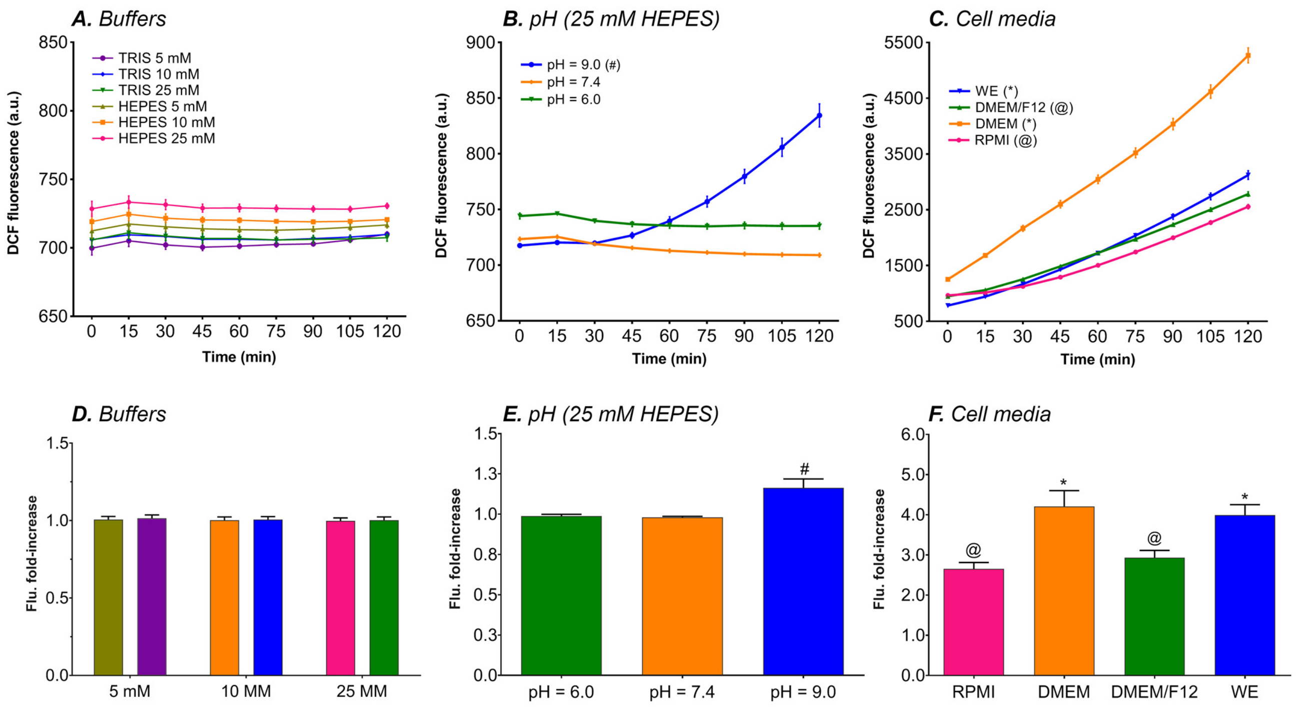

Figure S8. Influence of buffers (**A** and **D**), pH (**B** and **E**), and cell-culture media (**C** and **F**) on DCF formation under cell-free conditions (i.e., the data summarized in Table 2 of the main manuscript). DCF fluorescence was recorded for 2 h using a microplate reader and is presented as mean ± SEM for 8 values per condition. Additional experimental details are provided in the main manuscript (see *Section 3.1* and the Table 1 legend). The mean (± SD) fold-increase in DCF fluorescence over 2 h is presented in panels (**D-F**) for clarity. # indicates $p<0.05$ versus both other pH values, * indicates $p < 0.05$ versus all other culture media, and @ indicates $p < 0.05$ versus DMEM and WE. Statistical analysis was performed as described in the main manuscript (see *Section 2.6* and Table 2 legend).

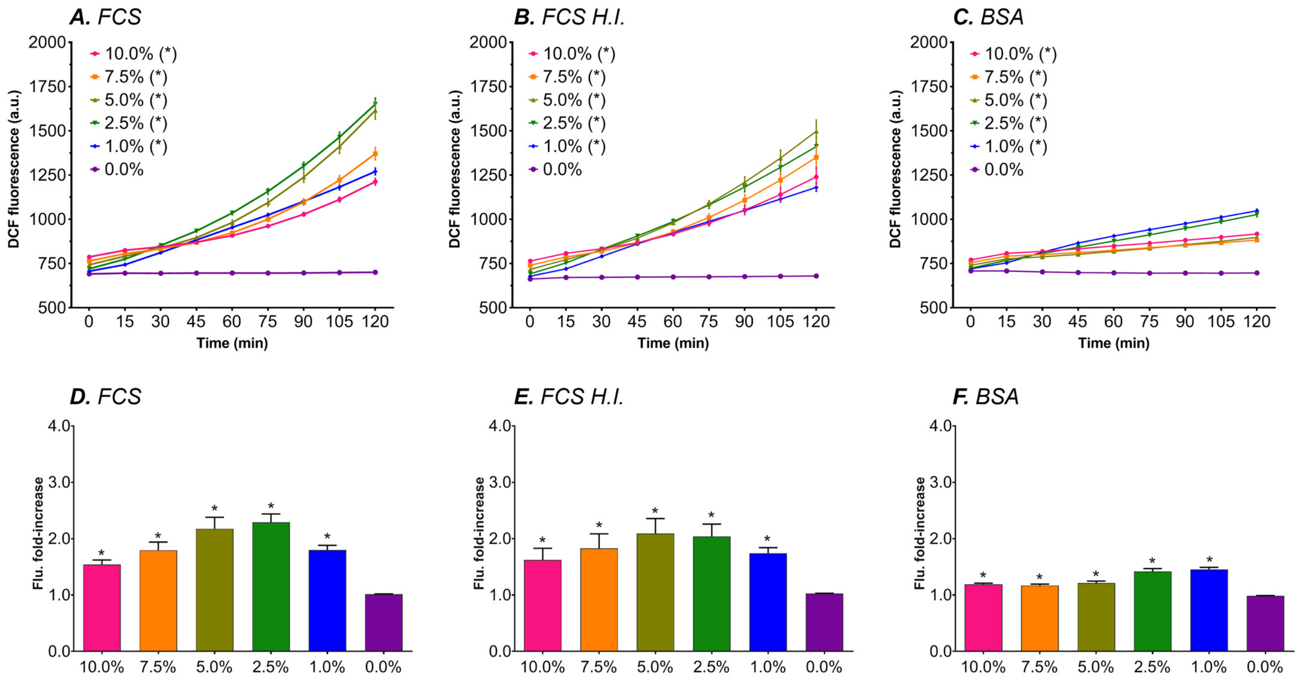

Figure S9. Influence of fetal calf serum (FCS, **A** and **D**), heat-inactivated fetal calf serum (**B** and **E**), and bovine serum albumin (**C** and **F**) on DCF formation under cell-free conditions (i.e., the data summarized in Table 2 of the main manuscript). DCF fluorescence was recorded for 2 h using a microplate reader and is presented as mean ± SEM for 8 values per condition. Additional experimental details are provided in the main manuscript (see *Section 3.1* and the Table 1 legend). The mean (± SD) fold-increase in DCF fluorescence over 2 h is presented in panels (**D-F**) for clarity. For all shown experiments, * indicates $p < 0.05$ versus the 0 μM (i.e., vehicle control) group. Statistical analysis was performed as described in the main manuscript (see *Section 2.6* and Table 2 legend)

Supplementary References

1. Bhogal RH, Weston CJ, Curbishley SM, Bhatt AN, Adams DH, Afford SC (2011) Variable responses of small and large human hepatocytes to hypoxia and hypoxia/reoxygenation (H-R). FEBS Lett 585 (6):935-941. doi:10.1016/j.febslet.2011.02.030
2. de Pedro N, Cautain B, Melguizo A, Vicente F, Genilloud O, Pelaez F, Tormo JR (2013) Mitochondrial complex I inhibitors, acetogenins, induce HepG2 cell death through the induction of the complete apoptotic mitochondrial pathway. J Bioenerg Biomembr 45 (1-2):153-164. doi:10.1007/s10863-012-9489-1
3. Zhang X, Zhou X, Chen R, Zhang H (2012) Radiosensitization by inhibiting complex I activity in human hepatoma HepG2 cells to X-ray radiation. J Radiat Res 53 (2):257-263. doi:10.1269/jrr.11124
4. Boyd J, Saksena A, Patrone JB, Williams HN, Boggs N, Le H, Theodore M (2011) Exploring the boundaries of additivity: mixtures of NADH: quinone oxidoreductase inhibitors. Chem Res Toxicol 24 (8):1242-1250. doi:10.1021/tx200098r
5. Cuello S, Goya L, Madrid Y, Campuzano S, Pedrero M, Bravo L, Camara C, Ramos S (2010) Molecular mechanisms of methylmercury-induced cell death in human HepG2 cells. Food Chem Toxicol 48 (5):1405-1411. doi:10.1016/j.fct.2010.03.009
6. Siddiqui MA, Ahmad J, Farshori NN, Saquib Q, Jahan S, Kashyap MP, Ahamed M, Musarrat J, Al-Khedhairy AA (2013) Rotenone-induced oxidative stress and apoptosis in human liver HepG2 cells. Mol Cell Biochem 384 (1-2):59-69. doi:10.1007/s11010-013-1781-9
7. Ryu HS, Park SY, Ma D, Zhang J, Lee W (2011) The induction of microRNA targeting IRS-1 is involved in the development of insulin resistance under conditions of mitochondrial dysfunction in hepatocytes. PLoS One 6 (3):e17343. doi:10.1371/journal.pone.0017343

8. Isenberg JS, Klaunig JE (2000) Role of the mitochondrial membrane permeability transition (MPT) in rotenone-induced apoptosis in liver cells. Toxicol Sci 53 (2):340-351. doi:10.1093/toxsci/53.2.340
9. von Montfort C, Matias N, Fernandez A, Fucho R, Conde de la Rosa L, Martinez-Chantar ML, Mato JM, Machida K, Tsukamoto H, Murphy MP, Mansouri A, Kaplowitz N, Garcia-Ruiz C, Fernandez-Checa JC (2012) Mitochondrial GSH determines the toxic or therapeutic potential of superoxide scavenging in steatohepatitis. J Hepatol 57 (4):852-859. doi:10.1016/j.jhep.2012.05.024
10. Farfan Labonne BE, Gutierrez M, Gomez-Quiroz LE, Konigsberg Fainstein M, Bucio L, Souza V, Flores O, Ortiz V, Hernandez E, Kershenobich D, Gutierrez-Ruiz MC (2009) Acetaldehyde-induced mitochondrial dysfunction sensitizes hepatocytes to oxidative damage. Cell Biol Toxicol 25 (6):599-609. doi:10.1007/s10565-008-9115-5
11. Busk M, Boutilier RG (2005) Metabolic arrest and its regulation in anoxic eel hepatocytes. Physiol Biochem Zool 78 (6):926-936. doi:10.1086/432857
12. Berthiaume F, MacDonald AD, Kang YH, Yarmush ML (2003) Control analysis of mitochondrial metabolism in intact hepatocytes: effect of interleukin-1beta and interleukin-6. Metab Eng 5 (2):108-123. doi:10.1016/s1096-7176(03)00010-7
13. Zhang JG, Nicholls-Grzemski FA, Tirmenstein MA, Fariss MW (2001) Vitamin E succinate protects hepatocytes against the toxic effect of reactive oxygen species generated at mitochondrial complexes I and III by alkylating agents. Chem Biol Interact 138 (3):267-284. doi:10.1016/s0009-2797(01)00278-2
14. Chen Q, Vazquez EJ, Moghaddas S, Hoppel CL, Lesnefsky EJ (2003) Production of reactive oxygen species by mitochondria: central role of complex III. J Biol Chem 278 (38):36027-36031. doi:10.1074/jbc.M304854200
15. Herrero A, Barja G (1997) Sites and mechanisms responsible for the low rate of free radical production of heart mitochondria in the long-lived pigeon. Mech Ageing Dev 98 (2):95-111. doi:10.1016/s0047-6374(97)00076-6
16. Votyakova TV, Reynolds IJ (2001) DeltaPsi(m)-Dependent and -independent production of reactive oxygen species by rat brain mitochondria. J Neurochem 79 (2):266-277. doi:10.1046/j.1471-4159.2001.00548.x
17. Degli Esposti M, Ghelli A, Crimi M, Estornell E, Fato R, Lenaz G (1993) Complex I and complex III of mitochondria have common inhibitors acting as ubiquinone antagonists. Biochem Biophys Res Commun 190 (3):1090-1096. doi:10.1006/bbrc.1993.1161
18. Keipert S, Ost M, Johann K, Imber F, Jastroch M, van Schothorst EM, Keijer J, Klaus S (2014) Skeletal muscle mitochondrial uncoupling drives endocrine cross-talk through the induction of FGF21 as a myokine. Am J Physiol Endocrinol Metab 306 (5):E469-482. doi:10.1152/ajpendo.00330.2013
19. Young TA, Cunningham CC, Bailey SM (2002) Reactive oxygen species production by the mitochondrial respiratory chain in isolated rat hepatocytes and liver mitochondria: studies using myxothiazol. Arch Biochem Biophys 405 (1):65-72. doi:10.1016/s0003-9861(02)00338-7
20. Shiryaeva A, Arkadyeva A, Emelyanova L, Sakuta G, Morozov V (2009) Superoxide anion production by the mitochondrial respiratory chain of hepatocytes of rats with experimental toxic hepatitis. J Bioenerg Biomembr 41 (4):379-385. doi:10.1007/s10863-009-9234-6
21. Nobes CD, Brown GC, Olive PN, Brand MD (1990) Non-ohmic proton conductance of the mitochondrial inner membrane in hepatocytes. J Biol Chem 265 (22):12903-12909
22. Johnson JE, Jr., Choksi K, Widger WR (2003) NADH-Ubiquinone oxidoreductase: substrate-dependent oxygen turnover to superoxide anion as a function of flavin mononucleotide. Mitochondrion 3 (2):97-110. doi:10.1016/s1567-7249(03)00084-9

23. Lambert AJ, Brand MD (2004) Inhibitors of the quinone-binding site allow rapid superoxide production from mitochondrial NADH:ubiquinone oxidoreductase (complex I). J Biol Chem 279 (38):39414-39420. doi:10.1074/jbc.M406576200
24. Choi WS, Palmiter RD, Xia Z (2011) Loss of mitochondrial complex I activity potentiates dopamine neuron death induced by microtubule dysfunction in a Parkinson's disease model. J Cell Biol 192 (5):873-882. doi:10.1083/jcb.201009132
25. Ohnishi ST, Shinzawa-Itoh K, Ohta K, Yoshikawa S, Ohnishi T (2010) New insights into the superoxide generation sites in bovine heart NADH-ubiquinone oxidoreductase (Complex I): the significance of protein-associated ubiquinone and the dynamic shifting of generation sites between semiflavin and semiquinone radicals. Biochim Biophys Acta 1797 (12):1901-1909. doi:10.1016/j.bbabio.2010.05.012
26. King MS, Sharpley MS, Hirst J (2009) Reduction of hydrophilic ubiquinones by the flavin in mitochondrial NADH:ubiquinone oxidoreductase (Complex I) and production of reactive oxygen species. Biochemistry 48 (9):2053-2062. doi:10.1021/bi802282h
27. Salaheldin TA, Loutfy SA, Ramadan MA, Youssef T, Mousa SA (2019) IR-enhanced photothermal therapeutic effect of graphene magnetite nanocomposite on human liver cancer HepG2 cell model. Int J Nanomedicine 14:4397-4412. doi:10.2147/ijn.S196256
